# Islam, mental health and law: a general overview

**DOI:** 10.1186/s12991-017-0150-6

**Published:** 2017-07-06

**Authors:** Georgios A. Tzeferakos, Athanasios I. Douzenis

**Affiliations:** 2nd Psychiatry Department, Attikon University Hospital, National and Kapodistrian University of Athens - Medical School, 1 Rimini Street, 12462 Athens, Greece

**Keywords:** Islam, Mental health, Forensic psychiatry, Mens rea

## Abstract

Islam is the dominant religion in about 56 countries around the globe, and has more than 1.2 billion followers. Islam represents a holistic way of life, and according to a large proportion of its followers, the Islamic law or Shari’ah should prevail over secular law and should be implemented as state law. The etymological root of the word Shari’ah can be traced back to the harsh life in the desert and it means “pathway to be followed” or “path to the water hole,” since the water was the basic element and preserver of life. At the dawn of its historical course and at its moral and ethical core, Islam introduced many interesting and innovative beliefs concerning the mentally ill. Islam underlines the moral necessity for the protection and care of the vulnerable individuals, as dictated by God himself. On the other hand, beliefs about “possession” and stigmatization influence the peoples’ attitude against and apprehension of mental disorders. This strange admixture is reflected upon the status of the mental health services and corresponding legislation found in the different countries of the Islamic world.

## Background

Islam is a monotheistic religion, and its founder is the prophet Muhammad, who was born in 570 A.D. Muhammad was a merchant who meditated in a desolate cave near Mecca, when at the age of forty he started listening to God’s speech (Allah), which was brought to him by Archangel Gabriel. This kept on for 23 years, until his death in 632 A.D. Due to the fear of political prosecution he moved from Mecca to Medina in 622 A.D. This event (hijra) marks the beginning of the Islamic calendar, which consists of 12 lunar months; it has 354 days and is not related to seasons [1]. In Medina, he founded the first community of his followers. The followers of Islam are called Muslims and they believe that the orders of their religion apply in every field of their life and they represent a complete life model [2]. Muslims are the majority in 56 countries, and it is estimated that they represent the one-fifth of the world population (more than 1.2 billions) [3].

## Legislation in the Islamic law

The Islamic law or Shari’ah or Qānūn-e Islāmī started taking shape by Abu Hanifah (699–767 A.D) through the need for social reorganization, which should be based in the concept of solidarity and sympathy against the corrupted governing of Umayyad (661–750 A.D) [4]. Shari’ah is an archaic Arabic word meaning “pathway to be followed” [5] or “path to the water hole” [6].

Shari’ah is applied in countries in the Middle East, Africa, and Asia [7]. Among these, Saudi Arabia applies its purest form. An interesting phenomenon, mainly resulting from the colonization of different parts of the globe by Western countries, is the coexistence within the same legal ambit of both secular and Islamic law [8]. In the beginning, the colonizing countries showed no interest in interfering or altering the local customary and tribal laws, unless they hindered their economic goals and expansionism [9]. But later in an effort to expand the implementation of their law on the indigenous populations, they tried to incorporate the customary—religious laws in a comprehensive legal system by three main methods: “the codification of customary or religious law; the application by state courts of unwritten customary or religious law in a fashion analogous to the common law and the creation or recognitions of informal or customary courts run by local leaders” [10]. Nigeria, a former British colony which became an independent federation in 1960, is a typical example of such a state. The northern part of the country is inhabited mostly by Muslims, while in the southern part the majority of the population is Christian. In the south, the legal system is based on the Anglo-Saxon common law, while in the north, a dual judicial system, consisting of both common and Islamic law, is applied [11]. Unfortunately, the country is devastated by clashes, fueled by religious hatred. In Egypt, Shari’ah controls specific areas of social life, such as marriage, inheritance, property rights, and applies to both Muslim and non-Muslim populations. As far as other issues are concerned, the common law is applied [12]. Dual judicial systems can be found even in EU member states. For almost one century, in the north-east area of Greece called Thrace, the Muslim citizens have the right to settle their legal matters, apart from the Greek civil courts, with the help of Islamic judges, the muftis [13]. The mufti in Greece has a tripartite role being an Islamic legal scholar, an Islamic judge (qâdi), and the religious leader of the Muslim citizens [14].

The legislation is formed by the scripts which are called Naṣṣ, and has a divine and not a human origin. The scripts of the Islamic law are [15]:

1. The Aḥkām of Qur’an (Koran), which is divided into 114 Suwar (chapters) and consists of about 6236Ᾱyāt (verses).
2. Hadith, which is the record of Muhammad’s life.

The length of the Suwar is uneven, with the shortest one (Al-Kawthar) containing only three verses while the longest (Al-Baqara) has 286 verses. The Suwar are divided into Meccan (87) or Medinan (27), depending on the location of revelation, that is before or after Hijra [16]. The Qur’an contains about 500 commands. Since Qur’an is the revelation of God’s will, these commands are to be strictly followed, without any hesitation or dispute. Some of these lines-commands have a direct and specific meaning, while others have a vaguer context. The clarification and the adaptation of the commands deriving from Qur’an and Hadith to the contemporary social, political, and historical conditions are called ijtihad. Ijtihad, practiced by the legal scholars of Islam, is based on strictly defined orders which are sources of law as well [17]. The most important of them are: (1) The Ijmā‛, which means legal consensus and (2) The Qiyās, which means analogy.

Hadith’s scripts, in contrast to those of the Qur’an, do not have the same stability, uniformity, and importance. From these scripts, the most important and highly accepted ones are called Sunnah. There are six recognized collections of Sunnah, which were written in the ninth and tenth century A.D, during the Sahih period [3].

With historical evolution, different prominent Islamic legal scholars developed different models of interpreting and applying the divine law, as depicted from the sacred scripts. These different approaches, collectively constituting the Islamic jurisprudence or Fiqh, finally evolved in the different schools or Maḏāhib of the Islamic law. In the early years of Islam, there were many Maḏāhib, but eventually they were consolidated to very few. The Sunnis have five Maḏāhib: (1) Mālikī, (2) Shāfi‛I, (3) Ḥanbalī, (4) Ḥanafī, and (5) Ẓāhirī, while the Shiites have three: (1) Ja’fari, (2) Zaydi, and (3) Ismaili [15, 18].

## Mental health in the Islamic world

The three basic trends for explaining the mental disorder, from antiquity till today, are [19, 20]: (a) The Organic approach, based on biology and pathophysiology, (b) the Psychological which examines and analyzes the intrapsychic processes and conflicts and (c) the Magical or Sacred which apprehends insanity through a supernatural and divine scope. These three components in the Islamic world are in a dynamic and ever changing balance. A balance between prejudice and social stigmatization on one hand and intense religious and moral commitment to support the weak on the other.

The Islamic world, in its early years, had a pioneering approach concerning mental health and psychiatry. The first psychiatric hospitals were founded in Arabic countries [21, 22]: Baghdad 705 A.D (during the kingship of the caliph El Waleed ibn Abdel Malek), Cairo 800 A.D, and Damascus 1270 A.D, whereas the first psychiatric asylum in the West Europe, the Bethlem Hospital in London, was founded in the thirteenth century [23]. Important figures of the Arabic medicine are Razis (860–932 A.D) and Avicennas (980–1037 A.D) [24]. They have rich authoritative work as for example the Kitab al-Hawi and the Al-Qanun fi al-tibb, which are multivolume medical books. They fought off superstition, which had already dominated the Christian world [25]; they adopted the Hippocratic organic psychiatry and they applied psychological methods of therapy [1].

Currently, there are more than 50 Islamic states, and therefore, it is difficult to simply describe the mental health services in these countries. Some of them provide advanced therapies [26], and they have a modern legal framework (Sudan 1998, Jordan 2002, Oman 1992), while in some others, the therapy of the psychiatric patients includes cautery, exorcism, and physical violence [1, 3]. Some of the Arabic countries either don’t have a specific legislation for mental health (Yemen, Saudi Arabia, United Arab Emirates, Bahrain) or the corresponding legal framework is out of date (Egypt 1944, Morocco 1959, Syria 1981) [12]. They do not have specialized training in forensic psychiatry and do not possess organized forensic psychiatric services [24]. The notion that mental disorder has a daemonological or divine origin is widespread in the Islamic world [1, 27, 28]. Many times people seek help from religious therapists, who use lines from the Qur’an as treatment. The social impact and influence of these therapists is so important that in some countries they have are incorporated in the national health care system [29].

Under the Islamic law, the therapeutic bond between a patient and a doctor is considered sacred. According to Shari’ah, human justice cannot force a doctor to reveal information entrusted to him/her by a patient. Some Islamic legal scholars argue that lying into a court in order to preserve the confidentiality of the therapeutic relationship cannot be considered a sin [2]. In any case, in Muslim trials, only the views and the opinions of Muslim psychiatrists are accepted [3].

Despite the importance that the Islamic law attributes to the confidentiality of the therapeutic bond, it is overridden in cases of suicide attempts [2]. Suicide is considered a very big sin, a type of homicide. In the West, during the middle Ages, the term that was used for referring to suicide was “self murder”, and only recently replaced by “suicide” [30]. Muslim religion strictly forbids it and the divine law considers suicide a very big crime [31]. Direct consequences of this perception are the scarce recording of suicide attempts as such, since this could lead to the prosecution of the patient, and also that the rates of suicide and attempted suicides cannot be reliably estimated in Muslim countries [1, 23]. Apart from suicide, other forbidden actions (similarly to Judaism and Christianity) include homosexuality, extramarital affairs, prostitution, and (unlike Judaism and Christianity) alcohol consumption [32].

## Forensic psychiatry and Islamic law

### Mens rea & criminal responsibility

The concept of *mens rea*, the guilty intention, is fully accepted in the Islamic law. There is no crime if there is no criminal intention. The significance of the subjective element of a criminal action (and not only of the result of this action) gradually emerged in the Western Europe through the “publication” of the criminal law, which reached its climax during Charlemagne’s kingship (768–814 A.D) [33]. In terms of the criminal’s intention, the criminal actions are divided into [16]: (1) ‛Amd, intentional, and (2) Khaṭā’, unintentional. There is also a third category which applies only in cases of homicide: (3) the Shibh al-‘amd that is the quasi-intentional homicide. All homicidal acts are punishable by death. But if the victim’s family decides to accept compensation and not to punish the murderer, then the latter is set free, unless there is a decision on behalf of the authorities for an additional punishment. On the contrary, if the family does not accept the compensation, then the judicial system cannot override this decision.

According to Shari‛ah, the lunatics (Majinum which also means teacher, wizard or prophet) have impaired judgment and will and so they cannot be held accountable for their actions. Insanity in the Arabic language is called Junūn and its etymology means “hidden” or “invisible.” This etymology derives from the belief that insanity-mental disorder is caused by the demonic possession of the patient from “invisible” or “hidden” spirits (jinn). In Arabic, the word “jinn” has many meanings, like shelter, shield, screen, fetus, and madness. According to the Islamic religion, the “jinn” is a supernatural spirit, which can take a human or animal form and can be either good or bad [34]. The demonological apprehension of mental disorder can be traced in many cultural settings: the archaic English word ilfig meant “mad” but also “affected by the elves,” thus reflecting the common belief of that time that madness was caused by supernatural deities [35].

### Insanity

There is tripartite classification of insanity in the Islamic law: (a) absolute or continuous, (b) intermittent, and (c) partial. In the case of intermittent insanity, it has to be proven that the mental disorder was active at the time of the criminal act for the defendant to be found not guilty by reason of insanity. Otherwise, if the disorder was in remission and not active, the perpetrator is fully responsible for his acts [17]. The similarity between the concepts of *lucida intervalla* or *intermission* of the Roman law [36] and *photeinon diallimaton* (“bright interims”) of the Byzantine law [37, 38] is obvious. As far as criminal responsibility is concerned, the Islamic law recognizes two other categories, similar to insanity: (1) the Dahish, which means “sudden confusion” or “perplexity” and (2) the ‛Atah, which means mental retardation or dementia [17].

### Involuntary admission

A pivotal issue addressed in the Islamic law, lying in the interface between law and psychiatry, is the concept of involuntary admission of mental health patients. According to the principle of Al-Hajjer, the state can undertake the financial management of a person’s fortune, if he does not manage it “properly.” By extending the application of this legal procedure, Shari‛ah accepts the necessity of involuntary hospitalization. Τhis necessity lies on the patient’s “need for therapy” (including patients with substance use disorders) and not on the criterion of dangerousness [2]. The corresponding legislation in the European Union countries is not homogenous: some countries use the criterion of dangerousness (Austria, Belgium, France, Luxembourg), while others use the combination of two criteria: dangerousness and need for therapy (Denmark, Finland, Greece, Ireland, Portugal, United Kingdom). Only three EU countries use exclusively the criterion of the need for therapy: Italy, Spain, and Sweden [39].

## Islam, Ancient Greek, and European law: results from cultural osmosis

Since the Greek civilization laid the foundation of the Western scientific thought, it is important to underline some of the similarities between Islam and ancient Greek medical and legal tradition.

As mentioned before, in the early years of Islam, the social attitude towards mental patients was that of compassionate care. According to Dols [40], “The development of the early Islamic hospital had coincided with the massive translation of early, primarily Greek, scientific works into Arabic.” This historical fact shifted the Arabic scientific medical thought towards the Galenic and Hippocratic medical doctrines, which were taught and practiced in the Hellenistic Near East. These doctrines emphasized the rational and scientific explanation of mental disorders, based on the Hippocratic humoral theory, thus preventing theology, superstitious, and demonology to easily infiltrate the domain of mental health [40].

The relationship between science, rational thought, and anthropocentric perception of the physical phenomena on one hand and superstitious, moralistic, and theological apprehension of the world on the other is characterized by a diachronic struggle for domination. This struggle affects all aspects of human civilization, including the laws. As we have seen in Islam, the laws are of divine origin and thus cannot be altered. We can trace a similar perception in many archaic societies, including ancient Greece [41], at least until the fifth century B.C. Lycurgus’ laws of ancient Sparta are included in the so-called *Megali Retra* (Great Clause), which is chronically set in 650 B.C. [42]. The name *Retra*, according to Lidell and Scott [43], means the agreement between the one who sets (God Apollo) and the one who accepts the laws (Lycurgus), while according to Plutarch [44], it is an oral declaration with divine origin.

Besides their divine origin, there are other similarities between ancient Greek and Islamic law: they both share a tripartite penal classification of homicide and also the provision of bloodwit or forgiveness in cases of homicide. Ancient Athenian penal law, based on Drako’s *phonikos nomos* (homicidal law), referred to the *mê ek pronoias* (unintentional), *ek pronoias* (intentional), and *akousios* (quasi-intentional) homicides [45, 46]. The criminal prosecution of the murderer could be suspended if the victim’s family accepted the bloodwit or if they granted *aidesis* (forgiveness) to the perpetrator [47]. The legal tradition of blood wit can be traced back to the pre-Homeric era. Homer in Iliad [48] (18th rhapsody, verses 497–508) described the scene of a trial, carved on the famous Achilles’ shield, forged by god Hephaestus himself, in the fires of mount Olympus. In front of the Agora of the city, where the most respected citizens consulted the king [49], the adverse parties presented their arguments about whether the bloodwit was paid or not. In another part of Iliad (9th rhapsody, verses 632–636), Ajax prompts Achilles to accept the compensation that Agamemnon offers him and in this way to forgive the insult of the abduction of his mistress Briseis.

From a forensic psychiatric point of view, an important historical question arises: did the notion of criminal insanity existed in the ancient Greek jurisprudence? According to Simon and Ahn-Redding [50], the theoretical foundations for this basic legal doctrine, found also in Islamic law, can be traced back in the ancient Greek philosophy. Plato [51] in his Laws introduces the innovative position that one should not be found accountable for his actions if he is mentally ill, elder, or juvenile. Despite this, there seems to be a scientific controversy about whether this philosophical conception was actually incorporated into the Athenian legislation or not [52, 53]. According to some researchers [50, 54, 55] insane criminals were treated in a more lenient way, while others [56] suggest that Plato’s Laws was a theoretical and philosophical thesis that does not necessarily reflect the legal status quo of classical Athens.

It was the Romans that adopted the doctrines of the Greek philosophy and transformed them into laws, thus creating the Roman legal system. The Roman Law was the cornerstone on which the common European legal tradition was forged [57]. This tradition, through colonization, influenced the legal systems of many countries, including Islamic ones. The law that stands at the foundation of the Roman legal system is the Lex Duodecim Tabularum, or the Law of the Twelve Tables. The composition and promulgation of the Lex Duodecim Tabularum were the result of the social struggle between the upper class of the Patricians and the lower class of the Pleibeians. The latter compelled the written codification of the customary law that was implemented and administered by their social rivals, thus limiting their power. The Lex Duodecim Tabularum contained statutes relating to both Jus Publicum that underwent extensive changes with the course of time, and Jus Privatum, which remained relatively unchanged and kept its fundamental role in the Roman jurisprudence [58–60]. Other Roman laws related to homicide, civil liability (and thus culpa), and criminal insanity were the Lex Aquila (286 B.C.) and the Lex Cornelia de sicariis et veneficis (82 B.C.) [61].

Although the legal foundations for the specialized penal treatment of the mentally disabled were laid in the Roman legal system, there is no historical proof that doctors appeared in courts giving testimonies as specialized experts. There is no citation of any kind of collaboration between legal scholars and physicians in the work of Galen of Pergamon (130–210 A.D.), the greatest physician in the Roman Empire, although Hippocratic medicine, with the help of the humoral theory, could explain different psychopathological conditions [62]. The European legal civilization had to go through the dark era of the Middle Ages, in order to find a convergence point with medicine. It was 1486 A.D. when the Dominican inquisitors Heinrich Kramer and Johann Sprenger published their notorious book Malleus Maleficarum (the witch hammer). This infamous work was a textbook that helped inquisitors to detect, prosecute and eliminate witches [63]. The result of this religious frenzy was that many mentally ill were accused of witchcraft and burned alive at the stake.

But, at the end of the sixteenth century A.D., while all this religious frenzy reached a climax, science started to fight back [64]. Two prominent doctors, Paracelsus or Theophrastus Bombastus von Hohenheim (1493–1541) and Johann Weyer (1515–1588), through their work supported the idea that mental disorders can be, also, explained and attributed to physical causes. It was the jurisdiction of medicine and not of theology to determine the cause of the different mental disorders [19, 64].

Another prominent physician, Paulus Zacchias (1584–1659), played a crucial role into the next evolutionary step of legal medicine and forensic psychiatry. This step consolidated the importance of the physicians’ expert opinion in different legal cases, thus paving the way for the establishment of forensic medicine and psychiatry. Zacchias has studied medicine and law at the Sapienza, became chief doctor of the Papal States, and he was advisor to the Rota Romana, the supreme Papal court of appeals [65]. He was the personal doctor of two Popes: Innocent X (1644-55) and Alexander VII (1655-67) [66]. He supported the idea that only doctors have the authority and the capability to evaluate a person’s psychopathological condition and he also introduced his own diagnostic system. In this system, he correlated and combined different mental disorders (like melancholia, phrenitis and insania) with legal terms and conditions (like fatui, ignorantes and stolidi) [67]. His most famous work was the Quaestiones medico-legales that consisted of nine libri and was published between 1621 and 1651. Although many historians and researchers cauterized and criticized him for endorsing tortures, suppression of women, belief in witchcraft, daemonology, and miracles, his work opened the way for medicine to establish a much upgraded status quo, not only in relation with legal science but generally in the human society [64, 65]. It is very interesting, from a historical, ethical, and sociopolitical point of view, to realize that a lot of prominent scientific figures that contributed to the beginning of the end of religious fanaticism came from ecclesiastical circles.

## Conclusions

In our study, we tried to give a general overview of the interconnection between Islamic faith, mental health, and law, focusing on specific key concepts and practices. In a world that is rapidly changing and big numbers of people move from one country to another, leading to cultural admixture, the necessity to familiarize with different cultures has become an imperative need. This study shows that despite big differences between western and Muslim societies, there are many common historical points, which could and should be the basis for a sincere and constructive dialogue. This necessity is of great importance in the field of Psychiatry and Forensic Psychiatry, where the subjective reality and identity of every person lies in a dynamic balance with the social, historical, and cultural ambit in which it lives.
